# Inhibition of the classical pathway of the complement cascade prevents early dendritic and synaptic degeneration in glaucoma

**DOI:** 10.1186/s13024-016-0091-6

**Published:** 2016-04-06

**Authors:** Pete A. Williams, James R. Tribble, Keating W. Pepper, Stephen D. Cross, B Paul Morgan, James E. Morgan, Simon W. M. John, Gareth R. Howell

**Affiliations:** The Jackson Laboratory, Bar Harbor, ME 04609 USA; School of Optometry and Vision Sciences, Cardiff University, Cardiff, CF24 4HQ UK; Institute of Infection and Immunity, School of Medicine, Cardiff University, Cardiff, CF14 4XN UK; Department of Ophthalmology, Tufts University of Medicine, Boston, MA 02111 USA; The Howard Hughes Medical Institute, Bar Harbor, ME 04609 USA; Graduate Program in Genetics, Sackler School of Graduate Biomedical Sciences, Tufts University, 136 Harrison Avenue, Boston, MA USA

**Keywords:** Glaucoma, Dendrite, Synapse, Complement, C1, C1qa, Retinal ganglion cell

## Abstract

**Background:**

Glaucoma is a complex, multifactorial disease characterised by the loss of retinal ganglion cells and their axons leading to a decrease in visual function. The earliest events that damage retinal ganglion cells in glaucoma are currently unknown. Retinal ganglion cell death appears to be compartmentalised, with soma, dendrite and axon changes potentially occurring through different mechanisms. There is mounting evidence from other neurodegenerative diseases suggesting that neuronal dendrites undergo a prolonged period of atrophy, including the pruning of synapses, prior to cell loss. In addition, recent evidence has shown the role of the complement cascade in synaptic pruning in glaucoma and other diseases.

**Results:**

Using a genetic (DBA/2J mouse) and an inducible (rat microbead) model of glaucoma we first demonstrate that there is loss of retinal ganglion cell synapses and dendrites at time points that precede axon or soma loss. We next determine the role of complement component 1 (C1) in early synaptic loss and dendritic atrophy during glaucoma. Using a genetic knockout of *C1qa* (D2.*C1qa*^-/-^ mouse) or pharmacological inhibition of C1 (in the rat bead model) we show that inhibition of C1 is sufficient to preserve dendritic and synaptic architecture.

**Conclusions:**

This study further supports assessing the potential for complement-modulating therapeutics for the prevention of retinal ganglion cell degeneration in glaucoma.

## Background

Glaucoma is a complex multifactorial disease characterized by the progressive dysfunction and loss of retinal ganglion cells and associated visual field deficits. It remains a leading cause of vision loss affecting 70 million people worldwide [1]. In spite of notable technical advances, the earliest neurodegenerative events that injure retinal ganglion cells during glaucoma [1–5] are unclear. Degeneration of the retinal ganglion cell during glaucoma appears to be partially compartmentalized, with different mechanisms influencing the soma, axon and dendritic tree. In human glaucoma, early changes to retinal ganglion cells have been observed both in the optic nerve head, including axon transport deficits [6–9], as well as in the retina with dendritic atrophy [10–12]. The precise relationship of axonal damage to events at the cell soma and retinal ganglion cell synaptic connectivity has been the focus of a number of recent studies in animal models of glaucoma [13–16] in which dendritic remodelling has been shown to precede gross cell loss. Recently, we demonstrated retinal ganglion cell dendritic atrophy during DBA/2J glaucoma and suggested that dendritic remodelling may be an early feature of glaucoma [16]. However, the mechanisms by which these early retinal ganglion cell changes occur are not known.

It has been proposed that activation of immune responses may be key events that damage retinal ganglion cells during early stages of glaucoma. Using a rat model of ocular hypertension (based on the sclerosis of episcleral vessels), Johnson et al. showed gene expression changes with significant enrichment of immune response pathways and cell proliferation pathways, among others [17]. The exploration of gene expression at the level of the optic nerve head confirmed immune cell responses and microglial activation, in the absence of significant astrocyte activation [18]. Guo and colleagues extended this work by showing an early immune cell response, coupled with gene expression patterns, that suggested retinal ganglion cell dysfunction and dendritic remodelling [19, 20]. Immune cell and/or microglial activation may therefore be a pertinent, early disease feature in animal models of glaucoma. Early changes to microglia [17–29] and peripherally derived immune cells [4, 30, 31] have been well documented in animal glaucoma models and in human glaucoma [32–34], consistent with this scenario of early immune activation.

The DBA/2 J mouse is a widely used model of chronic glaucoma showing hallmark features of the human disease. In DBA/2 J mice, mutant alleles of two genes (*Gpnmb*^*R150X*^ and *Tyrp1*^*b*^) cause iris pigment dispersion that results in ocular hypertension in the majority of eyes by 8 to 9 months of age. Glaucomatous damage is usually present by 12 months, signified by the loss of retinal ganglion cells and axonal degeneration within the optic nerve [35], which appears to initiate at the optic nerve head [2]. The interaction of multiple cell-types residing within the optic nerve head and retina are thought to be critical in glaucoma although the roles of specific cell-types are not well understood [3, 36–39].

We have previously used microarray gene expression of optic nerve head and retina tissue from pre-glaucomatous (8.5 months old) and glaucomatous (10.5 months old) DBA/2 J mice [3] in order to demonstrate that changes to inflammatory pathways, including the endothelin pathway, cell-adhesion pathways and the complement system occur early in the disease. In particular, induction of genes encoding proteins of the complement cascade was the earliest identifiable molecular change in glaucomatous DBA/2 J retinas [3]. DBA/2 J mice deficient for *C1qa* show robust protection from glaucomatous retinal ganglion cell loss and optic nerve degeneration [3]. The complement cascade has been heavily implicated in human and animal models of glaucoma, has increased expression in the eyes of patients with end stage glaucoma, and in primate and murine glaucomatous eyes [30, 40–49].

The role of the complement cascade in glaucoma is complex. In addition to its role in inflammatory signalling, complement pathways play a critical role in synaptic development and pruning [46, 50–53]. During central nervous system development neurons make many immature synaptic connections, followed by the selective elimination of those that are redundant. In the retinas of *C1qa*–deficient mice, retinal ganglion cells show defective synaptic elimination with limited refinement of the retinogeniculate pathway. TGF-β is thought to be important in the refinement process through activation/regulation of the complement cascade in neurons, microglia [51] or astrocytes [50]. The colocalization of C1q, part of the initiating complex of the classical pathway of the complement cascade, with synaptic markers such as PSD95 early in glaucomatous pathogenesis [50] supports the concept that complement activation underpins synapse elimination in glaucoma.

Here we determine the role of C1 in early synaptic loss and dendritic atrophy during glaucoma. We quantified synaptic and dendritic atrophy during early stages of glaucoma using two models of ocular hypertension. We demonstrate that synaptic and dendritic atrophy occur prior to any detectable damage to the axon or soma in both DBA/2 J glaucoma and in a rat model where ocular hypertension (OHT) was induced using an injection of magnetic microspheres [54]. Importantly, inhibiting C1 functions using either genetic ablation (DBA/2 J mutant for *C1qa*) or pharmacological inhibition (C1 esterase inhibitor in the rat OHT model) was sufficient to preserve dendritic and synaptic architecture. Taken together with the strong protective effect of *C1qa* knockout in retinal ganglion cell death and optic nerve damage, this suggests that inhibition of C1 should be considered as a therapeutic strategy for glaucoma.

## Methods

### Mouse strain, breeding and husbandry

Mice were housed and fed, as published [3], in a 14 h light/10 h dark cycle with food and water available *ad libitum*. All breeding and experimental procedures were undertaken in accordance with the Association for Research for Vision and Ophthalmology Statement for the Use of Animals in Ophthalmic Research. The Institutional Animal Care and Use Committee (IACUC) at The Jackson Laboratory approved this study. The DBA/2 J (D2, *n* (*mice*) = 24), DBA/2 J-*Gpnmb*^+^ (D2-*Gpnmb*^+^, *n* = 20), and D2.*C1qa* (*n* = 16) strains were utilized and have been described in detail elsewhere [3]. We used D2-*Gpnmb*^+^ mice as a control, non-glaucomatous substrain of DBA/2 J [55].

### Rat husbandry and IOP monitoring

Rat work was conducted in accordance with the Animals (Scientific Procedures) Act 1986. Brown Norway rats (Charles River, UK) were housed under a 12 h light/dark cycle with IOP measured at the same time of day in order to reduce variation in IOP. Breeding diet (SDS, RM3) and water were provided *ad libitum*.

### Glaucoma induction and IOP monitoring in the rat

Baseline IOP for both eyes was established prior to glaucoma induction and IOP measured 24 h after induction and every 3 days thereafter. IOP was measured using a rebound tonometer (TonoLab, Finland) calibrated for use on the rat eye. Animals were awake and unrestrained during measurement, requiring only topical 0.4 % oxybuprocaine hydrochloride eye drops (Midoptic, UK). IOP was taken as the average of 5 repeat readings. Glaucoma was induced as described for the magnetic microbead model [54]. Briefly, the left anterior chamber was injected with 10 μl of bead solution (sterile ~4.5 μm diameter paramagnetic microspheres, Invitrogen UK, in balanced salt solution) using a 32-gauge needle and Hamilton syringe (*n* = 19). Injections were conducted under isofluorane-induced anaesthesia with topical 0.5 % chloramphenicol (Mid Optic, UK) applied to the cornea. Beads were drawn into the iridocorneal angle using a small handheld magnet in order to block trabecular meshwork outflow and induce OHT (*n* = 20). The right eye remained an un-operated, normotensive (NT) control.

### C1 inhibitor administration

C1 inhibitor (C1 esterase inhibitor; CINRYZE; Shire (previously ViroPharma)) or vehicle only (PBS) was administered to rats 24 h prior to induced ocular hypertension (OHT) and then once every 4 days for 16 days. Intravitreal injections of 5 μl of C1 inhibitor (100 Units/ml (*n* = 17)) or PBS (*n* = 10) were performed on the left eye under isofluorane-induced anaesthesia.

### DiOlistic labelling of flat mount retinas

The protocol for retinal dissection and DiOlistic labelling was identical for both mouse and rat eyes except where stated otherwise. DiI and DiO coated tungsten bullets were prepared as described previously using 8 mg DiI and 16 mg DiO (both Life technologies, US) dissolved in 100 μl dichloromethane to 100 mg of tungsten particles (1.7 μm; Bio-Rad). Mice were killed by cervical dislocation, their eyes enucleated and retinas immediately dissected in chilled HBSS. For rats, a rising concentration of CO_2_ was used, with cervical dislocation confirming death. Retinas were dissected as described above. Retinas were then flat mounted ganglion cell layer (GCL) up on Millicell cell culture inserts (0.4 μm pore diameter, Life technologies, US). Retinas were then ballistically labelled with DiO/DiI bullets at a pressure of 120 psi using a Helios gene gun system (Biorad, US). 3.0 μm pore size cell culture inserts were placed between the gun and retina to prevent aggregated particles over labelling the retina. The culture inserts were then placed in Neurobasal-A media (Life technologies, US) containing 2 mM L-glutamate, 2 % B27 supplement and 1 % N2 supplement and incubated for 30 min at 37 °C, 4 % CO_2_ to facilitate dye diffusion. Retinas were then fixed in 4 % PFA for 30 min before mounting on glass slides with Fluoromount and coverslips.

### Immunofluorescent staining of retinal sections

The protocol for immunohistochemical staining of retinal sections was identical for both mice and rats except where stated otherwise. Mice were sacrificed by cervical dislocation, their eyes enucleated and placed in 4 % PFA ON. For rats, a rising concentration of CO_2_ was used, with cervical dislocation confirming death. Following this, eyes were cryoprotected in 30 % sucrose, frozen in OCT and cryosectioned at 12 μm. Eyes from the following number of animals (and central retina sections) were used: (in mice [animals (sections)]; 4mo D2 *n* = 6[34], 9mo D2 *n* = 3[25], 4mo D2-*Gpnmb*^+^*n* = 9[36], 9mo D2-*Gpnmb*^+^*n* = 5[28], 4mo D2.*C1qa n* = 5[29], 9mo D2.*C1qa n* = 3[30]. Rats: normotensive *n* = 9[43], normotensive plus C1 inhibitor *n* = 3[49], OHT *n* = 3[38], OHT plus C1 inhibitor *n* = 3[36]). Central retina sections were brought to RT for 1 h, permeabilized with 0.1 % Triton-X for 30mins, blocked with 5 % chick serum in PBS and stained ON at RT in primary antibody (for mice; rabbit anti-PSD-95; 51-6900, Life Technologies, USA, or goat anti-IBA1; ab5076, Abcam, UK; for rats; rabbit anti-PSD-95; ab18258, Abcam, UK). After primary antibody incubation, sections were washed 5 times in PBS, stained for 4 h at RT with secondary antibody (for mice; goat anti-rabbit AF488; Life Technologies, USA, for rats; goat anti-rabbit AF555, Abcam, UK). Slides were then washed a further 5 times with PBS, stained with DAPI (mice) or Hoechst 33342 (rats) for 15mins, mounted with fluoromount, coverslipped and sealed with nail-polish. For PSD95 quantification in DBA/2 J mice and rats, images of central retina showing the IPL and ONL were collected under identical conditions on a Leica SP8 confocal microscope and Leica DM6000B respectively (image area = 553.57 μm^2^). Fluorescent intensity of antibody staining was quantified using ImageJ. Three-dimensional reconstruction of IBA1+ cells was performed using IMARIS.

### Retinal ganglion cell morphological analysis

Retinas were imaged using either a Leica SP8 or Zeiss LSM 510 confocal microscope with ×20 objective (mice: 4mo D2 *n* = 63, 9mo D2 *n* = 58, 4mo D2-*Gpnmb*^+^*n* = 27, 9mo D2-*Gpnmb*^+^*n* = 37, 4mo D2.*C1qa n* = 30, 9mo D2.*C1qa n* = 52. Rats: normotensive *n* = 43, normotensive plus C1 inhibitor *n* = 49, OHT *n* = 38, OHT plus C1 inhibitor *n* = 36). Z-stack imaged (slice thickness 1 μm) were captured for mouse retinal ganglion cells and rat retinal ganglion cells ensuring that the entire dendritic tree was in frame. A number of RGC morphological features were measured using FIJI. Dendritic field area was measured by connecting the outermost dendritic tips using the convex polygon tool. The dendritic tree was traced and the mean dendritic length calculated using the Neuron J plugin. Sholl analysis was conducted using the bitmap Sholl analysis plugin for FIJI [56]. Statistical analysis was conducted using SPSS 20 (IBM, US).

### Classifying retinal ganglion cell subgroups

Retinal ganglion cells can be classified on the basis of their dendritic tree and soma morphology. To account for any bias that may arise from selective labelling of cells, we classified retinal ganglion cells from our control groups (in mice D2-*Gpnmb*^+^ and in rats, the normotensive group) based on classifications set out by Sun et al. [57, 58]. There was no obvious bias in the cell types expected (in mouse; our study (expected), RGC_A_ 9 % (10 %), RGC_B_ 38 % (30 %), RGC_C_ 41 % (37 %), RGC_D_ 13 % (23 %), in rat; RGC_A_ 20.9 % (17.5 %), RGC_B_ 32.6 % (29.2 %), RGC_C_ 34.9 % (35.2.3 %), RGC_D_ 11.6 % [18]). Once cells start to degenerate classifying based on morphology may lead to misrepresentation of cells at different stages of disease. Given that our control groups showed equal, unbiased sampling, we did not classify disease susceptible DBA/2 J or D2.*C1qa*^-/-^ retinal ganglion cells. As lamination is unlikely to drastically change during disease, we confirmed that an even proportion of ON/OFF/ON-OFF cells were present across our samples (in mouse; ON/OFF/ON-OFF, D2-*Gpnmb*^+^ 87 %/4 %/9 %, D2 86 %/7 %/7 %, D2.*C1qa* 86 %/6 %/8 %, in rat; normotensive 71 %/17 %/12 %, normotensive plus C1 inhibitor 74 %/12 %/14 %, OHT 82 %/7 %/10 %, OHT plus C1 inhibitor 84 %/8 %/8 %). This is further expanded on in the Discussion.

### Axon labelling with PPD and grading of glaucomatous damage

The processing of optic nerves and staining with paraphenylenediamine (PPD) which darkly stains the axoplasm and myelin sheath of damaged axons has been reported previously [59]. In brief, intracranial portions of optic nerves were fixed in 4 % PFA at RT for 48 h, processed and embedded in plastic. A segment of optic nerve from within a region up to 1 mm from the posterior surface of the sclera was sectioned (1 μm thick sections) and stained with PPD. Typically 30-50 sections are taken from each nerve. Homology between sections is considered during grading. Optic nerves were analysed and only eyes that had a corresponding nerve grade of ‘no or early damage’ (*i.e*. less than 5 % axons damaged) were selected in order to evaluate early disease events prior to axonal damage.

## Results

### Synapse reduction precedes optic nerve damage and is *C1qa* dependent

Dendrites degenerate prior to significant axon degeneration in DBA/2 J mice [16]. However, the factors that drive this dendritic atrophy in glaucoma are not known. Given the role of the complement cascade in synapse loss during development and neurodegenerative diseases [50], and the early induction of the complement components in the inner plexiform layer of glaucomatous retinas [3], we hypothesized that complement may mediate both synapse loss and dendritic atrophy in glaucomatous retinas. To test this, we first assessed the synaptic density of the inner plexiform and ganglion cell layer of 9 month-old DBA/2 J mice (an age at which IOP elevation is established) using a synaptic marker, PSD-95. To focus on very early stages of glaucoma, eyes were selected that had no detectable signs of glaucomatous axon damage (*see*Methods). Previous studies have shown that at 9 months, axonal transport is intact in these eyes [4, 16]. DBA/2 J eyes were compared to age matched control eyes (D2.*Gpnmb*^+^) and pre-glaucomatous DBA/2 J eyes (4 months prior to IOP increase). In both these control strains, at the ages selected, there was no detectable axon damage.

**Fig. 1:**
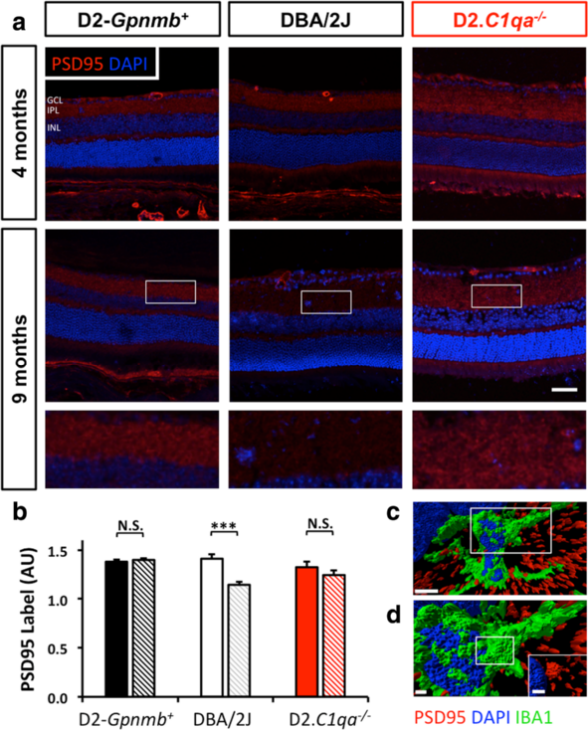
DBA/2 J mice exhibit synapse loss early during glaucoma pathogenesis which is absent in D2.*C1qa* ^-/-^ retinas. **a**. Immunohistochemical labelling (PSD-95, red) to demonstrate synaptic integrity in 9 months old DBA/2 J retinas. All eyes shown had no significant retinal ganglion cell or axon loss. Using *C1qa* mutant DBA/2 J mice (D2.*C1qa* ^-/-^) we explored whether synaptic pruning was present in these retinas. At 4 months D2.*Gpnmb* ^+^, DBA/2 J and D2.*C1qa* ^-/-^ retinas show comparable synapse density (**a**, *top row*). By 9 months, there is significant synapse loss in retinas of DBA/2 J mice that is absent in retinas of either D2.*Gpnmb* ^+^ or D2.*C1qa* ^-/-^ (**a**, *bottom two rows*). **b**. The degree of PSD-95 labelling is shown. The region of interest is marked (white boxes in upper row). **c**. Rendered confocal images show PSD-95 localisation within IBA1+ microglia in the IPL of DBA/2 J mice. Inset shows a higher resolution image of a region of interest (white rectangle, **d**). **d**. Inset from C showing PSD95 engulfed by IBA1+ microglia, inset within **d** (white rectangle) shows PSD95 and DAPI only. Scale bar = 50 μm (**a**), 5 μm (**c**) and 1 μm (**d** and inset), error bars = SEM, *** = *P* < 0.001, GCL = ganglion cell layer, IPL = inner plexiform layer, INL = inner nuclear layer

To test the role of complement activation during synapse loss in the IPL, we measured synaptic density using PSD-95. Twelve-micron sections were labelled with anti-PSD-95 antibody and DAPI (to demarcate the boundaries of the inner plexiform layer). At a young, pre-glaucomatous age (4 months old) there was no difference in labelling intensity in the IPL between D2.*Gpnmb*^+^ and DBA/2 J mice consistent with similar synaptic development in these genotypes. However, in 9 months old, DBA/2 J eyes with no discernible optic nerve disease or RGC loss there was a significant reduction in synaptic integrity (20 % compared to 4 months old DBA/2 J, *P* < 0.001 and 18 % compared to aged matched D2.*Gpnmb*^+^, *P* < 0.001) (Fig. 1). Given that *C1QA* expression increases in the IPL early in glaucoma [3, 50] and that DBA/2 J mice deficient in *C1qa* are protected from optic nerve degeneration, we tested the role of *C1qa* in synapse degeneration in DBA/2 J glaucoma. In D2.*C1qa*^-/-^ eyes, PSD-95 labelling intensity did not reduce significantly with age (6 % 4-9 months comparison, *P* = 0.26) (Fig. 1a) consistent with a role for *C1qa* in glaucomatous synapse elimination. There was no significant difference in outer plexiform layer (OPL) PSD-95 intensity (*data not shown*). Supporting a possible involvement of retinal microglia in early retinal ganglion cell synapse loss, high-resolution imaging combined with 3D reconstruction revealed high-resolution rendered images of IBA1+ microglia containing PSD-95 (Fig. 1c) supporting the concept that activated microglia phagocytose C1q tagged synapses in the early stages of DBA/2 J glaucoma.

### Early RGC dendrite loss is preserved in D2.C1qa^-/-^ retinas

We next explored whether complement C1q deficiency prevents dendritic atrophy in DBA/2 J glaucoma. Dendritic integrity was quantified in DiOlistically labelled retinas from 9 month-old DBA/2 J, D2.*Gpnmb*^+^, and D2.*C1qa*^-/-^ eyes with no detectable optic nerve damage. Two hundred and sixty seven cells were identified as retinal ganglion cells (confirmed by the presence of an axon running in the nerve fibre layer towards the optic disc) imaged at 40× and then compressed to give a 2D image for quantification. Dendritic architecture was assessed by total dendritic field area, total dendritic length, and dendritic complexity [60]. At 4 months of age, there were no significant differences in any of these phenotypes between mice of each genotype. By contrast, 9 month-old DBA/2 J mice had a significant reduction in retinal ganglion cell dendritic integrity compared to control D2.*Gpnmb*^+^ mice (significant reduction in dendritic field area (*P* < 0.01), and trending decreases in AUC and total dendritic length which do not reach significance), consistent with earlier work on non-glaucomatous retinas at 12 months of age [16].

**Fig. 2:**
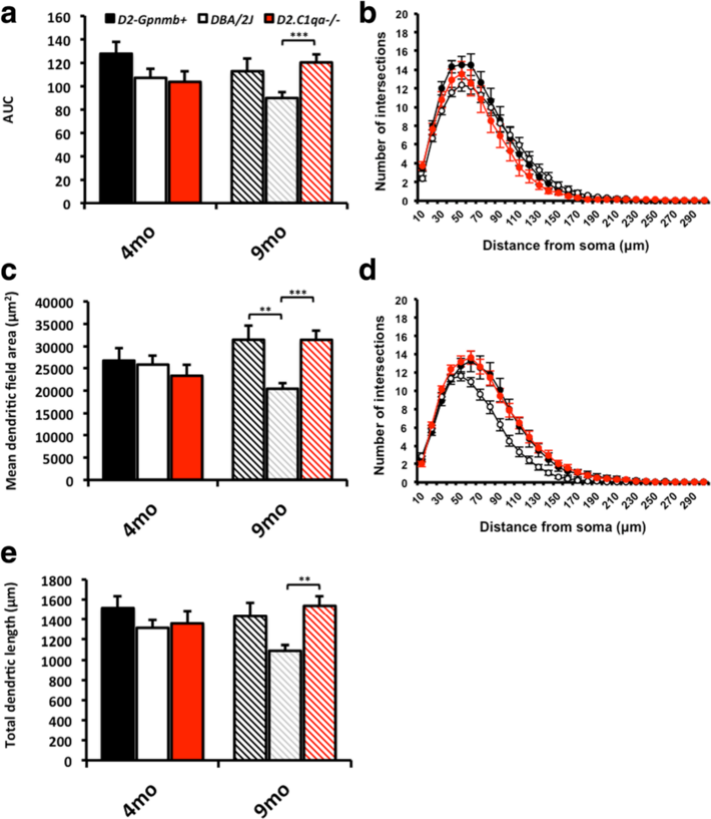
*C1qa* plays a role in dendritic pruning early during glaucoma pathogenesis. We tested whether *C1qa* has a role in dendrite remodelling using mice deficient in *C1qa* (D2.*C1qa* ^-/-^). **a** Area under the curve (AUC) of the Sholl analysis (at 4 months of age (**b**), and at 9 months of age (**d**)) shows a decrease in dendritic complexity in DBA/2 J retinal ganglion cells that is absent in D2.*Gpnmb* ^+^ and D2.*C1qa* ^-/-^ retinas. This is paralleled in both the retinal ganglion cell dendritic field area (**c**) and total dendritic length (**e**). Error bars = SEM, ** = *P* < 0.01, *** = *P* < 0.001

**Fig. 3:**
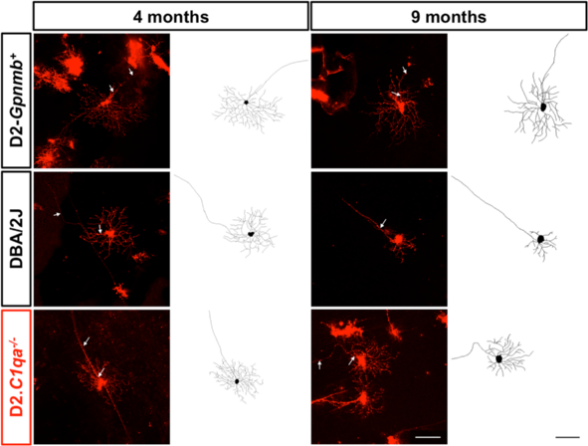
Representative DiOlistic and tracings of mouse retinal ganglion cells. Retinal ganglion cells from D2.*Gpnmb* ^+^, DBA/2 J and D2.*C1qa* ^-/-^ retinas were DiOlistically labelled to analyse dendritic morphology. *Top row*: representative images and tracings from 4 month (*left*) and 9 month (*right*) D2.*Gpnmb* ^+^ retinal ganglion cells. *Centre row*: representative images and tracings from 4 month (*left*) and 9 month (*right*) DBA/2 J retinal ganglion cells. *Bottom row*: representative images and tracings from 4 month (*left*) and 9 month (*right*) D2.*C1qa* ^-/-^ retinal ganglion cells. Scale bar = 100 μm, white arrows = axon

Dendritic atrophy was not present in D2.*C1qa*^-/-^ retinal ganglion cells, the dendritic trees of which were unchanged compared to the RGCs from D2.*Gpnmb*^+^ control mice (Fig. 2), confirming the critical importance of *C1qa* and its protein product C1q for early dendritic atrophy in DBA/2 J glaucoma. In addition there was no significant change in soma sizes across groups (*data not shown*). A panel of representative, DiOlistically labelled retinal ganglion cells is shown in Fig. 3.

### C1 inhibitor protects retinal ganglion cells from dendritic and synaptic atrophy

**Fig. 4:**
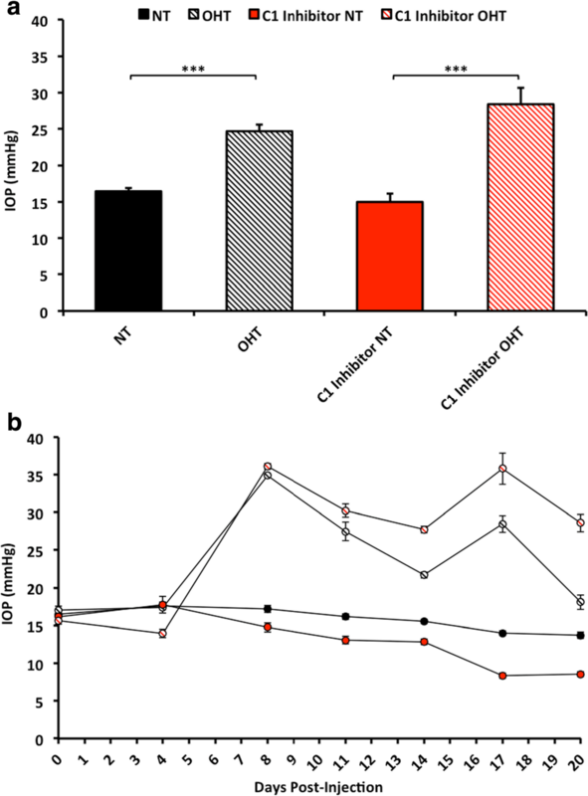
Ocular hypertension occurs in rat eyes treated with C1 inhibitor. **a** Average IOP over a 20 day time course. **b** Individual IOP recordings at each day of measurement (0 = day 0, base line reading). Points represent an average of 5 readings per eye. Note that C1 inhibitor administration does not significantly affect intraocular pressure. Error bars = StDev. *** = *P* < 0.001

**Fig. 5:**
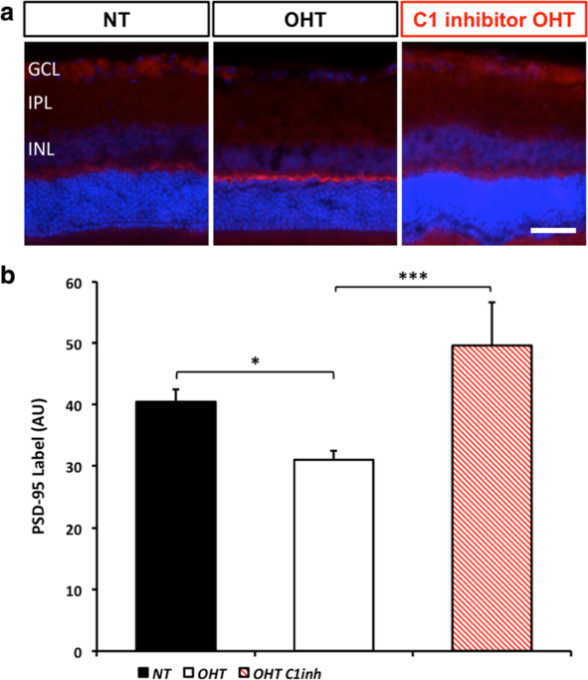
Ocular hypertensive rat eyes exhibit early inner plexiform layer synapse loss which is absent in C1 inhibitor treated eyes. Administration of a commercial FDA approved C1 inhibitor reduced synapse loss. **a** Representative images of rat retinas labelled with PSD-95 to show synapses. **b** Analysis of synaptic density. NT = normotensive eyes, sham control, OHT = ocular hypertensive eyes. Scale bar = 50 μm, * = *P* < 0.05, *** = *P* < 0.001, GCL = ganglion cell layer, IPL = inner plexiform layer, INL = inner nuclear layer

**Fig. 6:**
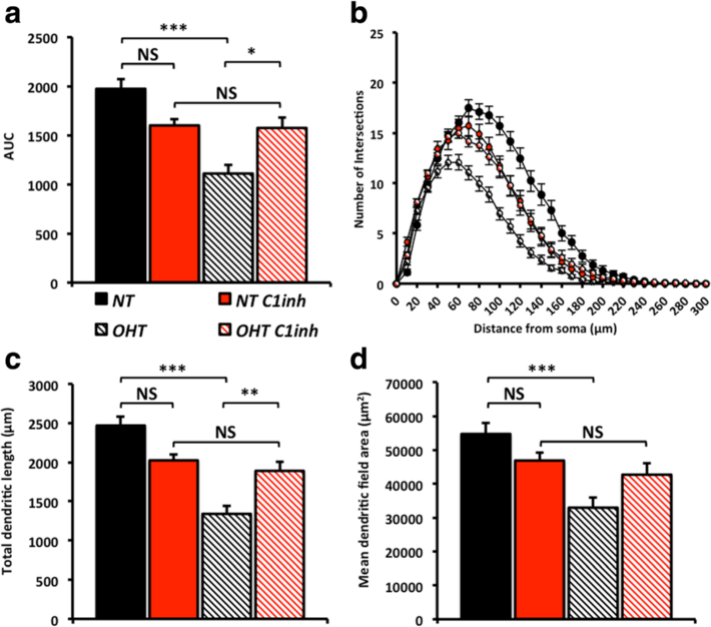
Inhibition of C1 prevents retinal ganglion cell dendrite pruning in ocular hypertensive rat eyes. To test dendrite loss in the rat bead model of glaucoma we DiOlistically labelled retinal ganglion cells following an ocular hypertensive insult. There was a dramatic decrease in dendritic integrity of retinal ganglion cells in ocular hypertensive eyes compared with normotensive eyes as assessed by Sholl analysis (**a**, area under the curve (AUC), **b**, Sholl analysis). This is paralleled in both the retinal ganglion cell dendritic field area (**c**) and total dendritic length (**d**). NT = normotensive eyes, sham control, OHT = ocular hypertensive eyes. Error bars = SEM, * = *P* < 0.05, ** = *P* < 0.01, *** = *P* < 0.001

**Fig. 7:**
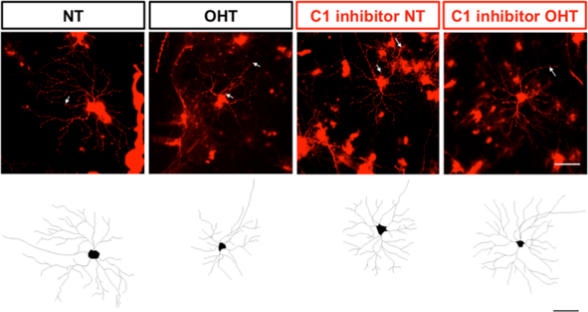
Representative DiOlistic and tracings of rat retinal ganglion cells. Retinal ganglion cells from normotensive eyes and ocular hypertensive eyes were DiOlistically labelled to analyse dendritic morphology. *Top row*: representative images and *Bottom row*: tracings from normotensive (*far left*), ocular hypertensive (*centre left*) normotensive (*centre right*), and ocular hypertensive (*far right*) retinal ganglion cells treated with C1 inhibitor. Scale bar = 100 μm, white arrows = axon

To further evaluate the role of C1q in dendrite and synapse integrity in glaucoma, we used an experimentally induced bead-model of ocular hypertension in the rat. In this model paramagnetic beads (~4.5 μm in diameter) are injected into the anterior chamber and pulled into the trabecular meshwork under the influence of an external magnetic field to generate sustainable increases in IOP [54] (Fig. 4). As with the DBA2J mouse, a reduction in PSD95 labelling indicated significant synapse loss in the IPL with retinal ganglion cell dendritic atrophy in glaucomatous eyes compared to the control, (un-injected), contralateral eyes (Figs. 5, 6 and 7). There was no significant difference in OPL PSD-95 intensity (*data not shown*).

To evaluate the role of C1q in synaptic loss and dendritic atrophy in the rat model, and to assess therapeutic benefit of pharmacologic inhibition of the C1 complex, we administered human C1 inhibitor intraviterally 1 day prior to the induction of ocular hypertension and then at 4 day intervals for a period of 28 days for those animals with sustained elevation in IOP. C1 inhibitor-treated eyes were significantly protected from RGC dendritic and synaptic atrophy compared to normotensive (NT) eyes. No significant dendritic or synaptic atrophy was observed in treated normotensive eyes (that received C1 inhibitor without OHT) (Figs. 5, 6 and 7). In addition there was no significant change in soma sizes across groups (*data not shown*). Importantly, the C1 inhibitor had no significant effect on the IOP profile (Fig. 4). Collectively, our data support a major role for C1 and the classical pathway of the complement cascade in dendritic and synaptic pruning during early glaucoma.

## Discussion

Multiple compartments of the retinal ganglion cell are suggested to be impacted in glaucoma [61]. While the optic nerve head is considered a primary and critical site of damage in glaucoma [2, 6, 8, 9, 62] early damage may also manifest at other locations including the retinal ganglion cell soma, dendrites and synapses [3, 10–12, 16, 63–67]. In this study, we use both a genetic (DBA/2 J mice) and an inducible (rat bead) model of glaucoma to show that significant changes occur to retinal ganglion cell synapses and dendrites prior to optic nerve degeneration. This dendritic and synaptic atrophy is disease- but not age-related as there is no detectable atrophy in age and strain matched, non-glaucomatous controls (D2.*Gpnmb*^+^). At this stage of the disease there is no damage as detectable by PPD staining of the optic nerve or retinal ganglion cell loss, predicting that observed effects [68] might be subtle. It cannot be ruled out that there was regional bias in the DiOlistic labelling in areas of the retina that are resistant to damage or contain retinal ganglion cells of varying morphologies. This seems unlikely given that DiOlistic labelling is diffuse (typically 5-20 cells per retina labelled evenly across the retina) and random. The preferential damage or loss of selective types of retinal ganglion cells has been investigated previously in the DBA/2 J mouse model, with neither cell type specific loss of dendritic morphology or somal shrinkage apparent [13, 69]. In addition, we have previous shown that genetic labelling of retinal ganglion cells introduces an inherent bias that might misrepresent cells at different stages of the disease in DBA/2 J glaucoma [16]. However, new, unbiased genetic tools that allow the visualization of specific retinal ganglion cell subtypes are being developed and provide scope for further investigation once available across mouse strains and in the rat [68]. These early changes to retinal ganglion cell structures in the retina are consistent with the early loss of pattern electroretinogram (PERG) that occurs in DBA/2 J mice [2, 70]. Since dendritic integrity is essential for the efficient generation of action potentials in the retinal ganglion cells, we suggest that dendritic degeneration may be an important contributor to the reduced PERG signal in early glaucoma [71, 72].

Determining the timing of changes to retinal ganglion cell compartments in the retina in relation to axonal changes in the optic nerve head in humans and animal models will be important to fully understand the earliest drivers of glaucoma. Although early soma and axon loss have been reported in 9 months old mice in other colonies [73], this is not the general case in our DBA/2 J colony [35]. However, in eyes with no significant optic nerve damage (based on optic nerve damage assessment just behind the orbit) or axon transport disruption, discrete and focal axon damage is observed in the glial lamina region of the optic nerve head at this stage [2]. The extent of the changes to retinal ganglion cell synapses and dendrites in the retina are greater than the number of focally damaged axons in the optic nerve head suggesting synaptic and dendritic atrophy are amongst the earliest pathological events in DBA/2 J glaucoma. To explore whether this is exclusive to the DBA/2 J mouse we also studied these changes in an inducible model of rat ocular hypertension, showing dendritic atrophy in eyes with consistent elevations in IOP but not in sham treated, normotensive or contralateral eyes.

Since early synaptic and dendritic atrophy were associated with an increase in complement components in the IPL [50] the complement pathway was a promising candidate for mediating early retinal ganglion cell degeneration. The key finding in the present study is that early synaptic and dendritic atrophy were prevented through inhibition of the C1 complex, the initiating complex of the classical complement pathway. D2.*C1qa*^-/-^ mice developed the anterior segment changes that occur in normal DBA/2 J mice with subsequent elevation in IOP [3]. Only a small percentage of D2.*C1qa*^-/-^ mice go on to develop optic nerve degeneration and retinal ganglion cell loss [3]. At 9 months of age (prior to any retinal ganglion cell loss and optic nerve degeneration) we could not discern any significant reduction in retinal ganglion cell dendritic integrity or synapse number compared to age matched, control D2.*Gpnmb*^+^ retinas, *i.e*. DBA/2 J dendritic and synaptic atrophy is prevented by *C1qa* deficiency. The functional implications of this protective effect (e.g. if the PERG is restored in DBA/2 J mice deficient in *C1qa*) have yet to be elucidated.

Given our findings in DBA/2 J glaucoma we sought to test whether pharmacological inhibition of C1 could be of therapeutic value in glaucoma. We demonstrate that intravitreal administration of human C1 inhibitor prior to and during the onset of OHT preserved dendritic and synaptic integrity in eyes with sustained elevations in IOP. Global genetic inhibition of C1 in the mouse protects all compartments of the retinal ganglion cell. It is important to note that, C1 inhibitor treatment in the rat did not protect the optic nerve, most likely reflecting the reduced accessibility of the drug outside of the retina (*data not shown*). This shows inhibiting complement in the retina is sufficient to protect dendrites and synapses even whilst axon degeneration is occurring. Our results further support compartmentalised changes in the retinal ganglion cell possibly due to independent, but not necessarily mutually exclusive, intraocular pressure-related events in the retina and optic nerve head. Studies employing the systemic administration of C1 inhibitors would clarify the protective effects of C1 inhibition in neural compartments outside the retina.

Complement activation has been shown in human and animal models of glaucoma [3, 49, 74], as well as in other chronic neurodegenerative diseases such as Alzheimer’s disease [75–82]. The classical pathway of the complement cascade is heavily implicated in glaucoma, and in DBA/2 J mice deficient in *C1qa* there is a significant decrease in retinal ganglion cell loss and optic nerve degeneration compared to regular DBA/2 J mice [3]. The complement cascade is activated by the classical, alternative or lectin pathways, converging at the cleaving of C3 into effector fragments that can modulate immune response such as chemo-attraction, phagocytosis or cell lysis. The precise mechanisms by which *C1qa* or the C1 complex contribute to glaucomatous retinal ganglion cell damage is not known but the opsonisation of synapses for removal by microglia [46, 50, 51, 53, 83], seems one likely route. C3-independent roles for the C1 complex have been also identified. For instance, C1q can bind apoptotic cells to promote their phagocytic uptake by myeloid-derived macrophages. C1q can also bind the frizzled receptor and modulate WNT signalling [84]. Components of the C1 complex are up-regulated in multiple cell types in the retina and optic nerve head during early stages of glaucoma, particularly myeloid derived cells and retinal ganglion cells. However, the extrinsic or intrinsic factors that induce or modulate complement induction are also not known. In the brain, *C1qa* expression increase in IBA1+ cells and neurons with age, and *C1qa* deficiency can mitigate age-related cognitive decline [53, 85]. Therefore, the complement cascade is playing multiple roles during different stages of glaucoma and further work is required to elucidate them.

## Conclusion

This study demonstrates in two different animal models of glaucoma that early synaptic and dendritic atrophy is dependent on C1QA, a component of the C1 complex of the complement cascade. It provides further evidence for the importance of complement activation in glaucoma, raising the possibility that complement-modulating therapeutics could play a role in the prevention of retinal ganglion cell damage.

## Abbreviations

ACUC: Animal care and use committee
AUC: Area under the curve
C1: Complement component 1
DiI - 1: 1'-Dioctadecyl-3,3,3',3'-Tetramethylindocarbocyanine Perchlorate
DiO - 3: 3′-dioctadecyloxacarbocyanine perchlorate
GCL: Ganglion cell layer
IBC: Institutional biosafety committee
OHT: Ocular HyperTension
IOP: IntraOcular pressure
IPL: Inner plexiform layer
NT: NormoTensive/NormoTension
OPL: Outer plexiform layer
RGC: Retinal ganglion cell
